# Termites Are Resistant to the Effects of Fire at Multiple Spatial Scales

**DOI:** 10.1371/journal.pone.0140114

**Published:** 2015-11-16

**Authors:** Sarah C. Avitabile, Dale G. Nimmo, Andrew F. Bennett, Michael F. Clarke

**Affiliations:** 1 Department of Ecology, Environment and Evolution, La Trobe University, Bundoora, Victoria 3086, Australia; 2 School of Life and Environmental Sciences, Deakin University, Burwood, Victoria 3125, Australia; Chinese Academy of Sciences, CHINA

## Abstract

Termites play an important ecological role in many ecosystems, particularly in nutrient-poor arid and semi-arid environments. We examined the distribution and occurrence of termites in the fire-prone, semi-arid mallee region of south-eastern Australia. In addition to periodic large wildfires, land managers use fire as a tool to achieve both asset protection and ecological outcomes in this region. Twelve taxa of termites were detected by using systematic searches and grids of cellulose baits at 560 sites, clustered in 28 landscapes selected to represent different fire mosaic patterns. There was no evidence of a significant relationship between the occurrence of termite species and time-since-fire at the site scale. Rather, the occurrence of species was related to habitat features such as the density of mallee trees and large logs (>10 cm diameter). Species richness was greater in chenopod mallee vegetation on heavier soils in swales, rather than *Triodia* mallee vegetation of the sandy dune slopes. At the landscape scale, there was little evidence that the frequency of occurrence of termite species was related to fire, and no evidence that habitat heterogeneity generated by fire influenced termite species richness. The most influential factor at the landscape scale was the environmental gradient represented by average annual rainfall. Although termites may be associated with flammable habitat components (e.g. dead wood), they appear to be buffered from the effects of fire by behavioural traits, including nesting underground, and the continued availability of dead wood after fire. There is no evidence to support the hypothesis that a fine-scale, diverse mosaic of post-fire age-classes will enhance the diversity of termites. Rather, termites appear to be resistant to the effects of fire at multiple spatial scales.

## Introduction

Fire is a widespread form of disturbance to environments throughout the world [1, 2]. Fire incinerates plant material, leading to changes in vegetation structure that can persist for decades or centuries [3, 4]. Over time, successive fire events influence landscape patterns by creating a mosaic of vegetation patches that differ with respect to their fire history [5]. In turn, these changes alter the distribution of animal species [6, 7], such that fire history is often a key driver of biodiversity patterns in fire-prone regions [8].

Termites (Isoptera) are common in fire-prone landscapes, including savannas worldwide [9, 10, 11] and arid and semi-arid woodlands [12]. Termites are ‘ecosystem engineers’ [13, 14], performing crucial roles in many ecosystems [14]. They contribute towards nutrient recycling and decomposition [15, 16], and affect the physical and chemical properties of litter and soil [17, 18]. Termites are also a major food source for animals (e.g. reptiles [19]; invertebrates [20]), and contribute to the formation of tree hollows, thereby providing shelter and breeding sites for many vertebrate species [21, 22].

Fire strongly affects habitat resources used by termites, such as plant biomass and dead wood [1, 3], and therefore may indirectly modify termite communities. However, there has been little research on the effects of fire on termites [9]. Most existing studies have been of short duration (e.g. < 3 years) and often confined to the period immediately following a fire (typically 1–3 years post-fire) [12, 19, 23]. Structural changes to vegetation following fire can continue for decades or centuries [3, 4, 24]; and consequently, short-term studies may overlook longer-term impacts of fire on animal species [25].

A further limitation is that existing studies generally have been conducted at a single spatial scale: the local or ‘site’ scale. These studies relate termite occurrence or community characteristics to the fire history at a particular *point* in the landscape [26, 27]. However, the response of species to their environment, and our ability to detect such responses, depends heavily on spatial scale [28, 29]. Thus, while site-scale studies provide valuable information regarding the effects of fire history on species occurrence at a local scale, they may overlook the effects of fire at broader spatial scales [30].

Both wildfire and fire management occur at a landscape scale, and create mosaics of fire-age classes that influence biodiversity throughout ‘whole’ landscapes (*sensu* [31], also see [7, 32, 33]). Fire management in regions around the world seeks to manipulate landscape patterns by creating a diverse mosaic of fire-ages under the assumption that greater spatial heterogeneity of fire-ages will enhance biodiversity (‘pyrodiversity begets biodiversity’) [5, 34]. Other landscape properties, such as the extent or spatial configuration of habitat, can influence individual species by affecting dispersal [35] and population size [36]. Few studies have examined how the properties of landscapes affect termites [37, 38], and none have done so in relation to the properties of fire mosaics.

The aim of this study was to examine the factors that influence the distribution of termites in a semi-arid, fire-prone region, over long temporal scales and at two spatial scales (site and landscape). First, we model how fire history affects the occurrence of termite species by using data from a 100 year, post-fire chronosequence of sites. Second, we examine the effects of habitat characteristics on termite species and on species richness of termites. As some habitat characteristics are influenced by time since fire and others are not [3], we predicted that the species most closely associated with fire-affected habitat characteristics would be those most strongly influenced by fire history at the site-scale. Third, we modelled the landscape-scale incidence of termite species and termite species richness in relation to the properties of entire fire mosaics, including the extent, configuration and composition of fire-ages within a landscape. Here, we predicted that species whose distributions were affected by fire history at the site scale would also be influenced by the properties of entire fire mosaics—specifically, their frequency of occurrence would be greater in landscapes with a larger spatial extent of their preferred fire-age [30, 33]—and that landscapes with a more diverse fire-history would contain a greater number of species [5].

## Methods

### Study area

The Murray Mallee region of south-eastern Australia encompasses an area of 104,000 km^2^ across three states (Victoria, New South Wales, South Australia). The climate is semi-arid with mean annual rainfall between 220–330 mm (Australian Bureau of Meteorology), high summer temperatures (mean daily maxima ≥ 32°C) and mild winters (mean daily maxima 16°C) [39]. Rainfall typically is non-seasonal and inter-annual rainfall variability is high. The dominant vegetation is ‘tree mallee’, characterised by multi-stemmed *Eucalyptus* species that occur as low shrubby trees (canopy height generally <5 m) and an understory of the hummock-grass *Triodia scariosa* or chenopod shrubs. Mallee vegetation is fire-prone. Large wildfires (e.g. >100,000 ha) occur regularly (~10–20 year intervals) [40], and current land management uses planned burning as a management tool [41]. Fires, both wildfire and planned burns, typically are ‘stand replacing’ with both canopy and ground vegetation removed and succession set to ‘year zero’ [3].

### Fire mapping

The fire age of study sites was determined by using one of two methods. For fires that occurred after 1972, fire scars were mapped across the study region using Landsat imagery from 15 individual years (1972–2007) [42]. Each fire scar boundary, and any unburnt patches within it, were digitized in ENVI 4.2. Digitized images were exported to ArcView 9.2 for data checking and to add attributes. When possible, exact fire years were attributed to fire scars using maps from land management agencies and local knowledge. For all other fire scars, we estimated fire age using the mid-point of the interval between images. For sites burnt before 1972, we used regression models to quantify the relationship between stem diameter and tree age for eucalypt species at sites of known age [43]. This was possible because fires in this system typically kill all above-ground vegetation, and mallee eucalypts subsequently re-grow new stems from underground lignotubers. The diameters of eucalypt stems therefore increase with the time since last fire [43]. The models we developed [43] were then used to estimate tree age, and infer fire-age, at sites of unknown fire age for which stem diameter data were collected. Validation of these models with independent data confirmed the strong correlation between actual and predicted age [43].

### Study design

We selected 28 landscapes, each a circular area of 4 km diameter (12.56 km^2^), across the study area. In the north, these were in Gluepot, Tarawi, Scotia and Danggali Reserves, and Lethero and Petro private reserves; and in the south in Murray-Sunset, Hattah, Billiatt, and Mallee Cliffs Reserves (see Figure 1 in [44]). The study landscapes were chosen to represent a gradient in the proportion (from 0–100%) of long-unburnt mallee vegetation (>35 years since fire) and variation in the number of post-fire age-classes present (from one to seven). The latter represents a measure of the heterogeneity of the ‘visible’ mosaic, those fire scars currently detectable in the landscape. Study landscapes were separated by a mean distance of 130 km (range: 6.3–217.7 km).

Termite surveys were undertaken at 20 sites within each landscape (n = 560 sites). The location of sites was stratified based on the proportion of each fire age-class in the landscape and, where possible, to encompass topographic variation (dune or swale, where a swale is the lowest point between dunes) within each fire age-class. Sites were at least 100 m from known fire boundaries, at least 25 m from a track and a minimum of 200 m apart. During November 2006, a large fire in Gluepot Reserve, South Australia, burnt 10 sites within landscapes 13 and 14; and so they had 10, rather than 20, sites surveyed. The geographic coordinates of all sites are listed in the supporting information (S1. Methods).

### Termite surveys

Two techniques for sampling termites were employed; active searches along transects and the use of cellulose baits. Active searches provided data on the termite species present and the microhabitats they occupy, but were costly in time and labour; therefore fewer sites were sampled than for baits. Data collected from active searches were used for both site- and landscape-scale analyses. Baits allowed a greater number of sites to be sampled across the study area but provided only presence/absence data for termite species for a site. Data from baits were used in landscape-scale analysis only. The data from the two survey methods (searches and baits) could not be combined for the site analysis due to the difference in sampling effort.

Active searches were carried out in spring (October and November) 2007, based on the method of Jones and Eggleton [45], but adapted for the semi-arid mallee ecosystem. Five 50 m x 4 m transects were sampled in each landscape (n = 140 transects in total). The transect was divided into 2 m x 5 m sections and within each section, all possible termite microhabitats were searched. This included the examination of soil cores (20 cores per transect, 10 cm^3^); all dead wood lifted, broken up and searched; hollow and dead branches removed from trees, and trees examined for nests to a height of 2 m. Ten pilot study searches established that a 50 m x 4 m transect consistently took two people one hour to complete (equivalent to two person-hours). Any termites found were recorded, along with the microhabitat type in which they were found. At each encounter, specimens of soldier and worker castes were collected into vials of 70% ethanol for later identification.

**Fig 1:**
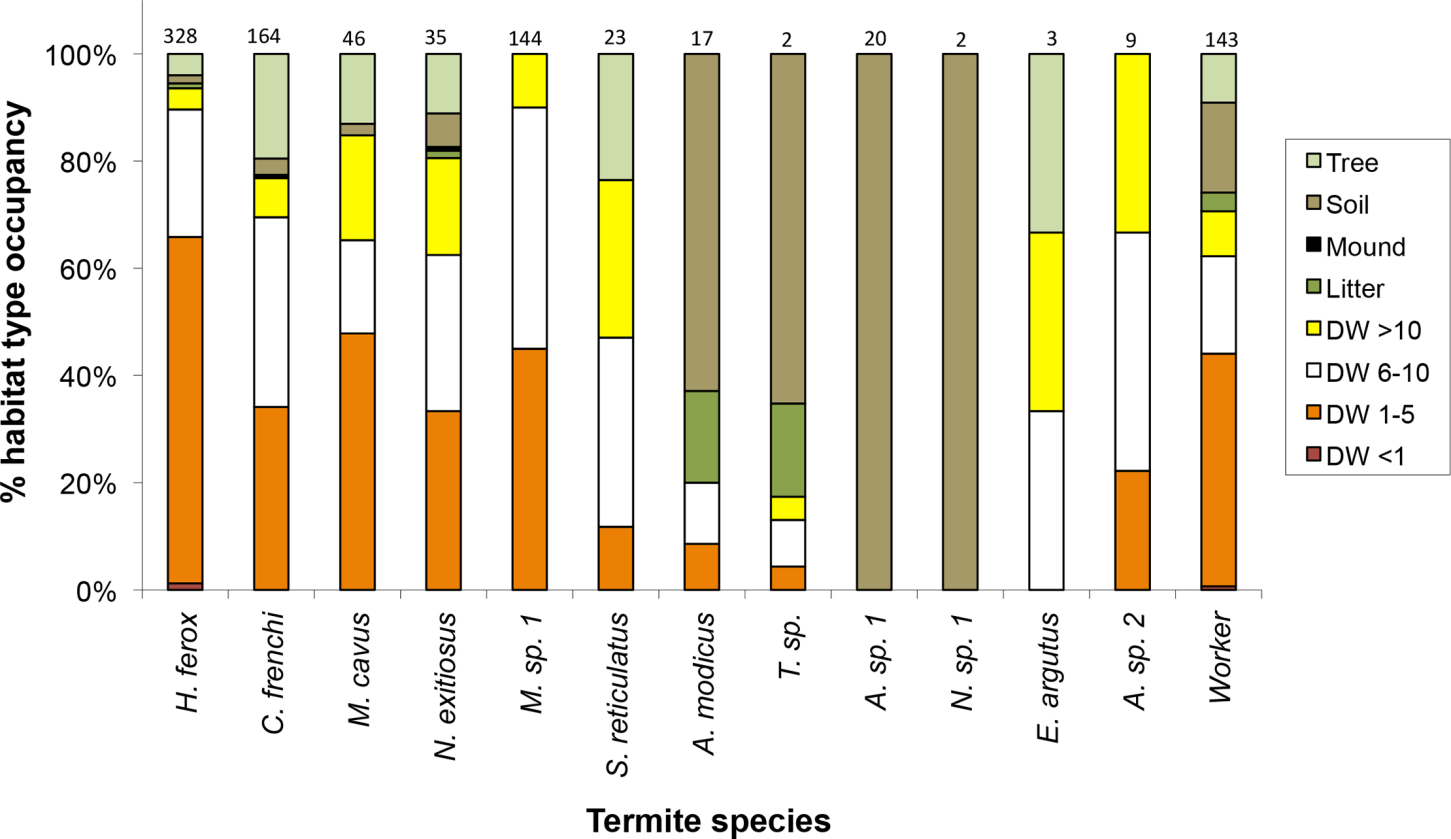
Habitat components in which each termite species was found. Shown as the percentage of records (total records at top of bars). DW <1 = dead wood less than 1 cm in diameter, DW 6–10 = dead wood 6–10 cm and DW >10 = dead wood greater than 10 cm diameter (n = 936 records from 140 transect searches).

Six cellulose baits (rolls of toilet paper; 400 sheet, two ply, bleached and unscented), spaced 5 m apart in a 3 x 2 grid, were buried in each of 20 sites, in each of the 28 landscapes (Fig 1). The toilet rolls were buried so that the top of the roll was approximately 2 cm below the surface of the soil. Each grid of six baits was considered an independent sample. Baits were installed in July/August 2006 and sampled in October/November 2006. During sampling, each bait was checked once by lifting the roll out of the ground and checking for termite presence. If baits had been disturbed (6.5% of 3360 rolls) but were still in place, then termites were collected if present (14% of disturbed rolls). Three sites had all 6 baits removed and were excluded from analysis. Soldier and worker castes were collected into a vial of 70% ethanol for later identification.

Termites were identified to genus and species where possible; with identifications confirmed by an independent expert (Mr Jim Creffield, formerly CSIRO Forestry and Forest Products).

### Explanatory variables

#### Site scale

A range of variables potentially influencing the distribution of termites was assessed at each site. Time since last fire (0–105 years) was determined for each site following the procedure outlined above. The long-term mean annual rainfall (1960–1990; Australian Bureau of Meteorology) was estimated from ABM models using data from the nearest weather station to each site (n = 7 weather stations used across the region; maximum distance of 100 km from site), and was included to account for a site’s location along the regional aridity gradient. Broad vegetation type for each site was determined by using maps developed by Haslem et al. [44]. These validated maps include three broad types of tree mallee vegetation, which vary in the dominant *Eucalyptus* species and understorey community: these are *Triodia* mallee, chenopod mallee, and heathy mallee.

Vegetation surveys were undertaken at each study site. Attributes of vegetation structure and ground cover were measured at 1 m intervals (points) along each 50 m line transect used for the active search and 10–15 m from bait sites. Vegetation structure was assessed by recording contacts with a 2 m vertical pole across four height strata (<0.5 m, 0.5–1 m, 1–2 m and >2 m). Ground cover at each point was classified (bare ground, litter, cryptogamic crust, plant, grass) and the proportion of each class was calculated. Litter depth was measured to the nearest cm at each 1 m point along the transect. The number of mallee (*Eucalyptus*) trees was recorded at each site within a 50 m x 4 m quadrat (overlaying the 50 m line transect), as were stem diameters (at 30 cm above ground) and the status of each stem (live or dead). The number of logs >50 cm in length was recorded in two size categories (>3 cm and >10 cm diameter) in a 50 m x 10 m quadrat (centred on the 50 m line transect). The topographic position of each site (dune, flat, swale) was also recorded.

A set of seven explanatory variables was calculated based on the vegetation surveys, and used to model the occurrence of individual species of termites at sites (Table 1). Five of these variables are influenced by the time since fire [3]: the number of large logs (>10 cm diameter), proportion of bare ground, maximum litter depth, average diameter of live mallee stems, and the structural complexity (proportion of possible ‘contacts’ by vegetation on a vertical pole in each height strata). Those variables not affected by time since fire included the presence or absence of *Triodia* and the density of trees (number ha^-1^).

**Table 1:** Description of explanatory variables used to model the distribution of termite species and assemblages at a) site and b) landscape scales in the Murray Mallee region.

*Variable*	*Name*	*Description*
a) Site scale		
Large logs	logs	Density of large logs >10 cm diameter (no. ha^-1^)
Bare ground	bare ground	% cover of bare ground
Litter depth	litter	Maximum litter depth (cm)
Presence of *Triodia*	Triodia	Presence/absence of the hummock grass *Triodia*
Diameter of stems	diameter	Mean diameter (cm) of live stems of mallee eucalypts
Structural complexity	str compl	Proportion of possible contacts with a 2 m ranging pole (n = 1300).
Tree density	tree	Number of mallee trees per ha (no. ha^-1^)
Landscape position	landsc pos	Dune crest, dune slope, flat or swale
Vegetation type	veg type	One of three vegetation types (Triodia mallee, chenopod mallee, heathy mallee)
b) Landscape scale		
Rainfall	rainfall	Average annual rainfall (mm) 1960–1990.
Extent of long unburnt	% old	Percentage area of landscape not burnt since 1972
Extent of recently burnt	% new	Percentage of area of landscape burnt since 1997
Extent of Triodia mallee	% Triodia	Percentage area of landscape covered by Triodia mallee vegetation type.
Diversity of vegetation types	veg diversity	Shannon's diversity index of vegetation types within the landscape
Diversity of fire ages	fire diversity	Shannon's diversity index of fire ages within the landscape

#### Landscape scale

The long-term mean annual rainfall (1960–1990; Australian Bureau of Meteorology) for each mosaic was used to represent the environmental and biogeographic gradient across the study area. The properties of the landscape were represented by variables that measured the extent and composition of landscape elements (Table 1). Extent variables included the percentage area of the landscape not burnt since at least 1972 (% long unburnt), the percentage of area burnt since 1997 (% recently burnt) and the percentage of the landscape covered by *Triodia* mallee vegetation (% *Triodia* mallee). Our analysis has shown that there are two broad vegetation types that cover the majority of the study area [44], one in which the understory is dominated by *Triodia* and the other by chenopod shrubs. The % area variables are highly correlated, therefore only one was used in the models.

Composition measures included the diversity (Shannon diversity index) of vegetation types (i.e. Triodia mallee, chenopod mallee and heathy mallee) based on the proportional area of each vegetation type in the landscape; and the diversity of fire age-classes (Shannon diversity index), which was characterized based on the proportional area of each fire age-class.

### Statistical analysis

All statistical analysis was undertaken in the R statistical package version 3.0.1 [46].

#### Site scale

The response variables at the site scale, using data from active searches only, were 1) the presence/absence of individual termite species, modelled assuming a binomial distribution of errors; and 2) termite species richness (i.e. the count of species recorded at a site), modelled assuming a Poisson distribution of errors. The response variables were chosen to explore effects on a) the occurrence of individual species of termite (i.e. tested by modelling presence/absence for individual species) and b) the species richness of termites (i.e. the total number of termite species at a site, a measure of community structure).

To investigate the responses of termites to time since fire, we used generalized additive mixed models (GAMMs). GAMMs allow explanatory variables to be fitted as linear or non-linear terms, and sources of correlation structure in the data can be included in models as random effects [47, 48]. Allowing for non-linearity was considered important when fitting fire response curves, as previous work has showed that species’ responses to fire can often be highly non-linear [25]. Time since fire and mean annual rainfall were entered as continuous fixed effects, and vegetation type was included as a categorical variable with two levels (*Triodia* mallee or chenopod mallee). An interaction between time since fire and vegetation type was specified using the ‘by’ command in the R package gamm4 [49]. These models allow response variables to have non-linear relationships with fire, which often occur [25], while also enabling a different relationship with time since fire in each vegetation type [50,51]. There were too few sites in ‘heathy mallee’ to fit a separate response to fire within this vegetation type (n = 12), and so these sites were omitted from this analysis. One additional site was excluded as it was considered an outlier with regard to time since fire (>105 years), resulting in 127 sites being included in this analysis. We are less confident of the accuracy of fire ages (from the estimation method) over 105 years, and this approach is consistent with analyses by the other authors in the Mallee Fire and Biodiversity Project [25, 50, 51].

Study sites were clustered within 28 landscapes. Consequently, for site-scale analyses, the landscape in which the site was located was included as a random factor to account for spatial structuring in the data due to this clustering of sites in landscapes (following [25, 51]). Smoothed terms were considered statistically significant when the p value was < 0.05; but, because p values for smoothed terms are approximated, values close to 0.05 were regarded with caution [48]. Model fit was evaluated using the percentage of null deviance explained (% dev). GAMMs were fitted using the package gamm4 version 0.2–1 [49].

To examine the effects of vegetation structure and ground cover on termites, we used generalised linear mixed models (GLMMs). As for GAMMs (above), GLMMs were used because they allow specification of both fixed and random effects [48]. Here, GLMMs were chosen in preference to GAMMs to avoid over fitting of models due to the larger number of predictor variables, and also to allow for model averaging of parameter estimates (which is not widely used for GAMMs). Prior to model fitting, we explored the relationships between response and habitat variables by using generalised additive models (GAMMs) to test for non-linear relationships; but such relationships were not evident. Explanatory variables were not strongly correlated (r <0.6) and were standardised (mean = 0, standard deviation = 1) to allow comparison of coefficients. Models (n = 128 models for each response variable) were developed for species that occurred at >10% of sites [25]. Model fit was measured by using both marginal and conditional R^2^ [52]. The marginal R^2^ represents the proportion of variance explained by the fixed factors (explanatory variables used in the models) alone, whereas the conditional R^2^ represents the proportion of variance explained by both the fixed factors and the random factors (here, the landscapes in which sites are clustered). GLMMs were fitted using the package MuMin version 1.9.5 [53].

We used an information theoretic approach to investigate the relative influence on response variables of seven site-level habitat variables chosen *a priori* as likely to influence the distribution of termite species: large logs, bare ground, litter depth, presence of Triodia, mean diameter of tree stems, structural complexity of vegetation, and tree density (descriptions given in Table 1). The information theoretic approach to model selection involves ranking alternative models according to the Akaike Information Criterion (AIC), with log likelihood employed as the measure of fit. Models with Δ_i_ <2 have substantial support from the data. The more models with Δ_i_ <2, the more uncertainty there is in identifying the best model. As no single model was clearly superior to others in the set (w_i_ <0.9) for any response variable, we used model averaging to determine the relative importance of explanatory variables [54]. An explanatory variable was considered influential if model averaging results showed that the 95% confidence interval of the estimate (coefficient) did not overlap with zero.

Finally, univariate GLMMs were fitted to examine the effects of broad vegetation type (*Triodia* mallee, chenopod mallee, or heath mallee) on presence or absence of termite species and on species richness of termites. Vegetation types vary in plant species composition, structure and soil type [45], and could influence termite distribution in the study area. These models were fitted separately to the habitat models due to strong collinearity between vegetation type and ground-cover variables.

#### Landscape scale

We used generalised linear models (GLMs) to investigate the response of termite species to landscape-scale explanatory variables. The response variables were species richness (total number of species found in a landscape) and species incidence (the number of sites at which a species was recorded), using data from both bait grids (n = 20 sites per landscape) and active searches (n = 5 sites per landscape). Models were fitted specifying a Poisson (for species richness) or binomial (presence/absence data) distribution of errors as appropriate. Where overdispersion was present, the adjusted quasi-likelihood AIC value (QAIC) was used.

We accounted for unequal numbers of sites surveyed in two landscapes (wildfire destroyed 10 bait sites in landscapes 13 and 14) by including a weight term (number of sites per landscape) in the binomial GLM and an offset term in the Poisson GLM. As with the site-scale analysis, non-linear relationships were examined (using GAMs) to assess whether a transformation of the explanatory variable would increase model fit. Explanatory variables were scaled (mean = 0, SD = 1) and only those with pairwise correlation coefficients < 0.6 were included. An information theoretic approach was used for model selection and model averaging. Model fit was assessed by using deviance explained (D^2^).

## Results

### Frequency of occurrence of termite species

Termites were detected at all 140 sites at which active searches were conducted, resulting in 793 identified termite collections (Table 2). In total, twelve species were recorded. *Heterotermes ferox* sens. lat. (Frogatt) (Rhinotermitidae) was the most frequently encountered species (41% of the samples). Two others, *Coptotermes frenchi* sens. lat. (Hill) (Rhinotermitidae) and *Nasutitermes exitiosus* (Hill) (Termitidae), were found relatively frequently (21% and 17% of samples), whereas most other species were found infrequently. Three species, *Nasutitermes* sp. 1, *Ephelotermes argutus* (Hill) (Termitidae) and *Amitermes* sp 2. (Termitidae), were found during these searches but were not detected with the bait grids.

**Table 2:** Species of termite found in mallee vegetation by using baits and active searches; the number of records (searches: encounters of termites, baits: presence on grid of six baits), and the percentage of sites and landscapes in which each species was found.

	Number of records		% of sites		% of landscapes	
Species	Searches	Baits	Searches	Baits	Searches	Baits
			(n = 140)	(n = 560)	(n = 28)	(n = 28)
Rhinotermitidae						
* Heterotermes ferox*	328	417	76	74	100	100
* Coptotermes frenchi*	164	30	56	5	100	54
* Schedorhinotermes reticulatus*	17	2	11	<1	36	7
Termitidae						
* Nasutitermes exitiosus*	144	16	31	3	75	36
* Nasutitermes* sp. 1	2	0	1	0	7	0
* Microcerotermes cavus*	46	22	24	4	71	50
* Microcerotermes* sp. 1	20	28	11	5	32	50
* Amitermes modicus*	35	40	12	7	39	61
* Amitermes* sp. 1	2	5	1	<1	7	18
* Amitermes sp*. 2	9	0	5	0	21	0
* Tumulitermes* sp.1	23	15	11	3	36	43
* Ephelotermes argutus*	3	0	2	0	10	0

In total, 88% of bait grids (i.e. n = 537 sites with one grid of six toilet roll baits per site) either had termites present when checked or showed evidence that termites had previously been present, such as casings or holes in the paper. Overall, 37% of baits were attacked by termites (total, n = 3222 baits). This technique yielded 575 identified termite collections from 537 bait grids. Nine species were collected and *H*. *ferox* was again the most common, accounting for 72% of all termites collected from baits. The next most common was *Amitermes modicus* (Hill) (Termitidae), from 7% of baits. Two species were found much less frequently on the baits than in searches: *C*. *frenchi* (5% *c*.*f*. 21%) and *N*. *exitiosus* (3% *c*.*f*. 17%).

### Use of habitat components by termites

During active searches, termites were found in a variety of habitat components, with dead wood being the most commonly occupied (Fig 1). Larger pieces of dead wood (>6 cm diameter) lying on the surface were the most species-rich habitat components, with all but two species found there (Fig 1). Only *H*. *ferox* and unidentified workers (most likely to be *H*. *ferox*) were found in the smallest pieces of dead wood (<1 cm). Some species were generalists and found in all habitat components (e.g. *N*. *exitiosus*, Fig 1) and others were found only in dead wood or litter on the ground (e.g. *Microcerotermes* sp.1). *Amitermes modicus* and *A*. sp. 1 were similar both morphologically and in habitat component preference and have been grouped together as *Amitermes* spp. in the following analyses.

### Factors influencing termites at the site scale

Time since fire did not significantly affect the distribution of any termite species, or the species richness of termites (Table 3). Several models of the response of individual species to time since fire failed to converge due to inadequate data across fire ages within the two vegetation types. Therefore, only a subset of species (n = 4) were modelled in this phase of analysis (Table 3).

**Table 3:** Summary of generalised additive mixed models of the species richness of termites and the presence/absence of individual species of termites in relation to time since fire, rainfall and vegetation type (CM = Chenopod Mallee; TM = Triodia Mallee) at the site scale. edf = estimated degrees of freedom.

Species	Vegetation type	Smoothed term for time since fire		
		edf	F	P
Species richness	CM	1	0.00	0.97
	TM	1	0.10	0.75
*H*. *ferox*	CM	1	0.03	0.87
	TM	1	0.53	0.47
*C*. *frenchi*	CM	1	0.15	0.70
	TM	1	0.47	0.50
*M*. *cavus*	CM	1	0.34	0.56
	TM	1	3.57	0.06
*N*. *exitiosus*	CM	1	0.35	0.55
	TM	1	0.56	0.45

**Fig 2:**
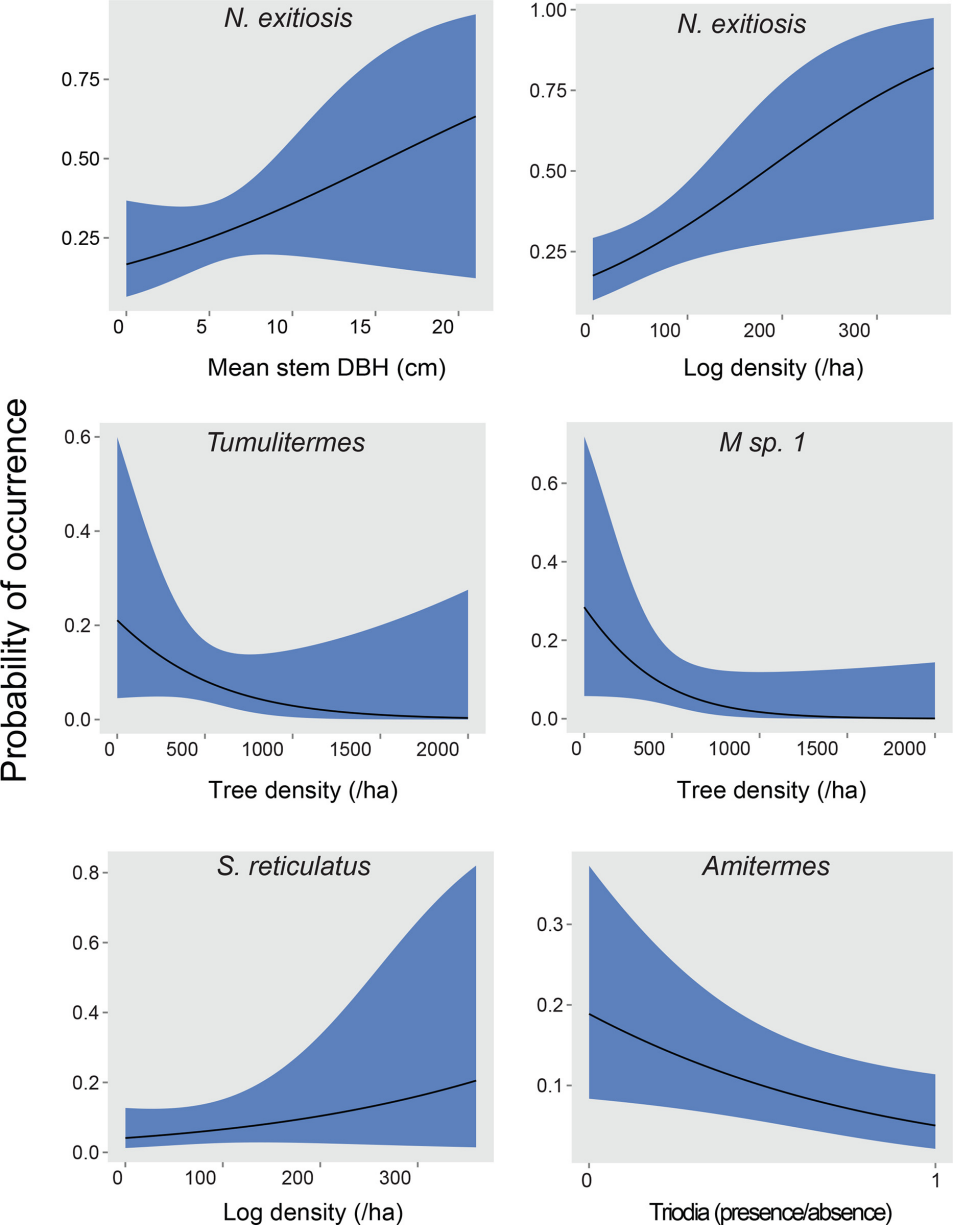
Responses of termite species to important explanatory variables at the site scale. Variables include density of trees, number of large logs, diameter of eucalypt stems and presence of *Triodia scariosa*. Solid lines represent predicted probability of occurrence from generalised linear mixed models, and shaded blue area represents ± 1 SE.

For eight species, there were sufficient data to model in relation to habitat structure, vegetation type and topographic position (Tables 4 and 5). The best fitting models were for *Amitermes* spp. and *N*. *exitiosus*. There was a greater probability of occurrence of *Amitermes* spp. at sites without *Triodia* hummocks and with less structural complexity (Table 5, Fig 2): *Amitermes* spp. were more abundant at sites in chenopod mallee vegetation (Table 4). Sites with greater tree density, larger stems and more logs were more likely to have *N*. *exitiosus* present (Table 5, Fig 2). This species was more abundant at sites in swales in chenopod mallee vegetation rather than heathy mallee and Triodia mallee vegetation (Table 4). *N*. *exitiosus* was predominantly found in dead wood on the ground (80% of samples, Fig 1).

**Table 4:** Summary of generalised linear mixed models of termite species occurrence at the site scale in relation to vegetation type (CM = Chenopod Mallee; TM = Triodia Mallee; HM = Heathy Mallee). HM was removed from the model when a species was absent at all sites in this vegetation type. Coefficients of explanatory variables (and standard errors) shown in bold are those for which the 95% confidence intervals of coefficients did not include zero. Chenopod mallee is used as the reference category of vegetation.

Species	Vegetation type		
		Coef.	SE
Species richness	TM	**-0.25**	**0.12**
	HM	**-0.68**	**0.25**
*Heterotermes ferox*	TM	0.79	0.43
	HM	1.03	0.84
*Coptotermes frenchi*	TM	0.40	0.41
	HM	-0.76	0.75
*Nasutitermes exitiosus*	TM	**-1.74**	**0.47**
	HM	-1.61	0.91
*Microcerotermes cavus*	TM	0.18	0.46
*Amitermes* spp.	TM	**-2.29**	**0.65**
*Tumulitermes* sp. 1	TM	-0.41	0.67
*Schedorhinotermes reticulatus*	TM	**-1.78**	**0.64**
*Microcerotermes* sp. 1	TM	-1.08	0.72

**Table 5:** Summary of generalised linear mixed models of the occurrence of termite species at the site scale. Coefficients of important explanatory variables (and standard errors) shown in bold are those for which the 95% confidence intervals of model-averaged coefficients did not include zero. Marginal and conditional R^2^ values indicate the model fit of the full model (all variables). Descriptions of explanatory variables are given in Table 1.

	trees		diameter		logs		str compl		bare ground		Triodia		litter		Marg.	Cond.
	Coef	SE	Coef	SE	Coef	SE	Coef	SE	Coef	SE	Coef	SE	Coef	SE	R^2^	R^2^
*Heterotermes ferox*	**0.67**	**0.29**	-0.28	0.23	0.27	0.25	0.23	0.25	0.19	0.29	0.05	0.50	-0.03	0.22	17	17
*Coptotermes frenchi*	0.31	0.20	-0.10	0.21	0.09	0.20	0.14	0.21	0.09	0.23	0.17	0.44	-0.18	0.19	5	11
*Microcerotermes cavus*	0.44	0.24	0.37	0.28	0.00	0.25	-0.32	0.26	0.17	0.33	0.05	0.57	0.33	0.22	12	21
*Amitermes* spp.	-0.03	0.34	0.15	0.33	-0.65	0.43	**-0.99**	**0.49**	-0.57	0.45	**-2.02**	**0.72**	0.48	0.35	40	50
*Nasutitermes exitiosus*	**0.79**	**0.26**	**0.55**	**0.26**	**0.68**	**0.24**	-0.37	0.30	-0.35	0.36	-0.45	0.59	0.16	0.23	31	33
*Tumulitermes* sp.1	**-1.05**	**0.54**	-0.27	0.40	-0.45	0.48	-0.09	0.38	-0.27	0.44	0.37	0.83	0.32	0.33	25	44
*Schedorhinotermes reticulatus*	-0.21	0.43	0.46	0.35	**0.60**	**0.32**	-0.44	0.46	-0.54	0.52	-0.74	0.82	-0.43	0.41	19	42
*Microcerotermes* sp. 1	**-1.35**	**0.63**	-0.30	0.43	0.03	0.33	-0.07	0.40	-0.25	0.48	0.91	0.86	0.07	0.34	32	47
Species richness	0.08	0.06	0.07	0.06	0.07	0.06	-0.07	0.06	-0.04	0.08	-0.13	0.13	0.03	0.06	6	6

In contrast, the presence of the most common species, *Heterotermes ferox*, was strongly related to fewer variables, with only density of trees having a positive relationship (Table 5, Fig 2). This species was found in all landscape positions (dune crests and slopes, clay or sandy swales) and all vegetation types. It also was found in all habitat components except mounds, but predominantly in smaller pieces of dead wood (Fig 1).

Species richness of termites was not related to any of the habitat variables included in the models (Table 5), but was higher in chenopod mallee vegetation (Table 4).

### Factors influencing termites at the landscape scale

The total number of termite species detected in study landscapes ranged from two species (Landscape 17, Billiat Conservation Park) to 10 species (Landscape 28, Lethero Reserve), including data from both bait grids and active search methods. Two species, *H*. *ferox* and *C*. *frenchi*, were recorded in all 28 landscapes; and five others. *M*. *cavus*, *A*. *modicus*, *N*. *exitiosus*, *T*. sp. and *M*. sp. 1, were found in more than 50% of landscapes. The remaining four species, *A*. sp. 1, *N*. sp. 1, *E*. *argutus* and *A*. sp. 2 were found in less than 20% of landscapes (Table 2).

For individual species, mean annual rainfall was influential for four species based on model averaging results (Table 6). The frequency of occurrence of *H*. *ferox* was positively associated with mean annual rainfall, and the full model explained 43% of the deviance. By contrast, the frequency of occurrence of *Amitermes* spp., *Tumulitermes* spp. and *S*. *reticulatus* were negatively associated with mean annual rainfall, with the models accounting for 30%, 35% and 33% of the deviance for these taxa, respectively. For these taxa, no other variables included in the models (other than rainfall) were influential as judged by model averaging. For the remaining termite species, diversity of vegetation types was influential for *C*. *frenchi*, extent of long unburnt mallee (> 35 years since fire) for *M*. *cavus*, and *N*. *exitiosus* was more likely to be found in landscapes with lower percentage cover of *Triodia* mallee (Table 6). For species richness of termites, none of the variables included in the model were identified as influential during model averaging (Table 6).

**Table 6:** Summary of generalised linear models for termite species at the landscape scale. Coefficients of important explanatory variables (and standard errors) shown in bold are those for which the 95% confidence intervals of model-averaged coefficients did not include zero. D^2^ values indicate model fit of the full model. Descriptions of explanatory variables are given in Table 1.

	Rainfall		% old		% new		Extent TM		Veg diversity		Fire diversity		D^2^ (%)
	Coef.	SE	Coef.	SE	Coef.	SE	Coef.	SE	Coef.	SE	Coef.	SE	
*Heterotermes ferox*	**0.43**	**0.14**	0.35	0.14	0.08	0.15	0.03	0.14	0.14	0.15	0.16	0.14	43
*Coptotermes frenchi*	0.05	0.12	0.07	0.13	0.14	0.12	0.08	0.13	**0.24**	**0.12**	0.09	0.12	35
*Microcerotermes cavus*	0.13	0.15	**0.34**	**0.15**	-0.29	0.21	0.10	0.16	-0.05	0.16	-0.19	0.17	39
*Amitermes* spp.	**-0.55**	**0.21**	-0.12	0.22	0.14	0.20	-0.10	0.20	0.21	0.24	0.12	0.22	30
*Nasutitermes exitiosus*	0.09	0.18	-0.11	0.18	-0.25	0.22	**-0.39**	**0.16**	0.19	0.18	0	0.21	30
*Tumulitermes* sp. 1	**-0.70**	**0.28**	0.35	0.27	-0.15	0.3	-0.01	0.30	-0.05	0.31	0.13	0.27	35
*Schedorhinotermes reticulatus*	**-0.69**	**0.21**	0.34	0.21	-0.14	0.24	0.01	0.24	-0.08	0.23	0.15	0.21	33
*Microcerotermes* sp. 1	-0.20	0.30	-0.48	0.30	-0.26	0.34	0.69	0.36	-0.48	0.3	0.04	0.33	27
Species richness	-0.10	0.08	0.06	0.08	-0.10	0.09	0.05	0.08	0.04	0.08	0.02	0.08	28

## Discussion

Termites are critical to ecological function in fire-prone regions throughout the world, and yet little is known about how termites are affected by fire [9]. We found that although termite species are found in association with above-ground habitat components potentially affected by fire (e.g. dead wood, litter), there were few strong associations between the occurrence of species and the relative abundance of such habitat characteristics that are vulnerable to fire. As a consequence, termites are largely resistant to the effects of fire at both the site and landscape scales. This resistance to fire means that the diversity of fire-ages within a landscape is not a useful surrogate for termite diversity.

### Relationships at the site-scale

Despite the substantial changes that occur to above-ground vegetation following fire in mallee ecosystems [3], fire history (time since fire) had no effect on any termite species or on termite richness, in any vegetation type. This finding lends support to recent studies which have concluded that termites generally are resilient to fire when considered over long time periods [26, 55, 56]. One explanation for the lack of response to fire in this study concerns the ecological traits of the species in our study region. Differences in responses to fire across termite species have been attributed to different feeding or nesting behaviours; for example, harvester [57] or wood-nesting species [10] have been more consistently affected by fire than mound-building [58] or subterranean species [26]. The termite fauna of the mallee region are predominantly subterranean nesting species. Subterranean colonies are buffered during fire by their position in the soil, as temperature increases are unlikely to penetrate more than 10 cm below the surface [59], and galleries and chambers may be as deep as 10 metres [60]. Other subterranean-nesting fauna, such as ants, have also been found to be resilient to fire [61, 62]. Thus, termites in mallee ecosystems may either not suffer high mortality during a fire event, or are buffered such that some colony members survive and populations can recover rapidly, therefore reducing the initial impact of fire on termite populations.

Although nesting below ground might buffer termites against fire, it is possible that termites could still be affected over the long-term due to the effects of fire on habitat or food resources (i.e. woody material). However, the habitat characteristics that most strongly influenced termite species are either not affected by fire (tree density), or are only weakly affected (log density) [3]. Individual mallee trees are resistant to fire because they resprout from a below-ground lignotuber that is buffered from fire events [63]. This means that fire does not affect the density of mallee trees at a site. While the density of logs is related to fire history, the relationship is relatively weak (~10% deviance explained; [3]). Importantly, Haslem et al. [44] showed that although log density is temporarily reduced in recently burned *Triodi*a mallee, log density increases rapidly (as tree stems killed by fire collapse) and remains at ~50% of the peak density even immediately following fire. Further, while fire may reduce food availability by incinerating woody material, it may also increase food availability if charred wood is as likely, or more readily, colonised than unburnt wood [64]. Sufficient food is therefore likely to remain post-fire to support termite colonies that survive at a site, either above ground as burnt or charred wood or below ground as lignotubers, roots and stems.

Vegetation type has been identified as an important variable influencing termite species and communities [26, 55]. In this study, most species did not differ between vegetation types, but three species had a preference for chenopod mallee and this vegetation type also had higher species richness. It is possible that the three vegetation types in the mallee do not differ in ways that greatly influence habitat for termites; for example, *Eucalyptus* trees dominate the canopy in both Triodia and chenopod mallee. There is an increase in the clay content of the sandy soils of chenopod mallee, compared with Triodia mallee and heathy mallee, which may influence the suitability for tunnelling for some species. Further investigation is required to understand the distributional patterns observed in this study.

### Relationships at the landscape scale

Landscape structure, at the scale studied, had little effect on termite species or species richness. Only one species was related to a fire-related variable at the landscape-scale; *M*. *cavus* was positively associated with the extent of long-unburned vegetation. This lack of relationships contrasts with contemporaneous research on reptiles [30], small mammals [33] and birds [65], which found some properties of fire mosaics to be important determinants for a broad array of individual species; and with Taylor et al. [7], who found that bird species richness was greatest in landscapes with a higher proportion of long unburned (>35 years since fire) vegetation. Species within each of those taxa also displayed significant relationships with fire history (i.e. time since fire) at the site scale [25, 50, 51], and responses to the properties of fire mosaics largely reflected these. For instance, the Desert Skink *Liopholis inornata* was most common in recently burned sites [51], and was negatively associated with the extent of long-unburned vegetation at the landscape-scale [30]. By contrast, no termite species responded to fire history in the current study. If termite species are not affected by fire history at a local scale, it is not surprising that the properties of fire mosaics do not drive species occurrence or community richness at larger scales.

It cannot necessarily be assumed that *all* relationships between species and their environment observed at the local scale will ‘scale-up’ to form similar relationships at the landscape scale. For instance, the spatial extent of *Triodia* mallee vegetation influenced (negatively) only one termite species (*N*. *exitiosus*), despite three species having negative associations at the site scale. This highlights that some relationships for species are scale dependent [28, 66]. It is possible that a relationship with vegetation type at the site scale (*Amitermes* with chenopod mallee, for example) may not have been strong enough to be detected at the landscape scale, due to the smaller sample size at the landscape scale (28 landscapes vs 140 sites) and the associated reduction in statistical power. Alternatively, while the scale of the landscape used in this study (4 km diameter) may be relevant for vertebrates (reptiles, mammals, birds), a meaningful ‘landscape’ scale for termites may be much smaller. Nevertheless, there is much value in multi-scale studies, as important relationships could be overlooked during single-scale studies when relationships are scale-dependent.

The diversity of fire-ages did not increase the landscape-scale species richness of termites. That is, pyrodiversity did not enhance termite diversity (*sensu* [5]). A lack of support for the pyrodiversity hypothesis was also found for birds [7], small mammals [33] and reptiles [67] in this same study system. Studies of termites in other ecosystems have also found limited support for the pyrodiversity hypothesis. For instance, Davies et al. [26] found that termites in African savannahs were resistant to a range of fire regimes, and so increasing the diversity of fire histories would not enhance termite diversity. Collectively, these studies question the wisdom of burning to achieve a fine-scale patch mosaic of diverse fire-ages in the hope that it will enhance biodiversity.

The landscape variable with the strongest influence on termites was the location of the landscape along a climatic gradient, represented by mean annual rainfall. Although there was no relationship with species richness, three of eight species had a negative relationship with rainfall at this scale and one had a positive relationship. Globally, the generic diversity of termites decreases with increasing latitude, with tropical rain forests generally having the highest richness [68]. In Australia, the pattern differs whereby savanna woodlands and semi-arid ecosystems have greater richness than tropical rain forests [68]. Why this is the case requires further study.

## Conclusions

Current fire regimes have no detectable and lasting influence on the occurrence of termite species or the species richness of termite assemblages in the semi-arid mallee environment. Rather, these taxa appear to be resistant to the effects of fire, probably due to their behavioural traits of nesting underground and the continued presence of dead wood in the post-fire environment. The occurrence of individual species was more closely related to habitat components, such as the density of mallee trees and large logs. Three of eight species favoured chenopod mallee vegetation that typically occurs on heavy soils of the swales between dunes, and species richness also was greater in this vegetation type. Termites play a key role in ecosystem function in mallee ecosystems, particularly through their role in decomposition and nutrient cycling. Given their ubiquitous distribution and resistance to fire, it appears that these functional roles are not likely to be affected either by wildfire or planned burning in this ecosystem. Further work is required to understand the factors that determine the distribution of species and structure of termite communities, and that regulate their role as an ecosystem engineer in this semi-arid environment.

## Supporting Information

S1 Dataset Termite site scale data used in analysis. (XLS)

S2 Dataset Termite landscape scale data used in analysis. (XLS)

S1 Methods Geographical coordinates of sites. (XLS)
